# Supplementary material for: Substituted Syndecan-2-Derived Mimetic Peptides Show Improved Antitumor Activity over the Parent Syndecan-2-Derived Peptide
Source: Int J Mol Sci. 2022 May 24;23(11):5888. doi: 10.3390/ijms23115888 (PMC9180903; doi:10.3390/ijms23115888)
Supplement: Supplementary file 1 [file ijms-23-05888-s001.zip › ijms-1683827-supplementary.pdf]

Original images are shown below. Partial images used in the manuscript has been marked in square boxes.

A

Original gels presented in Figure 1A

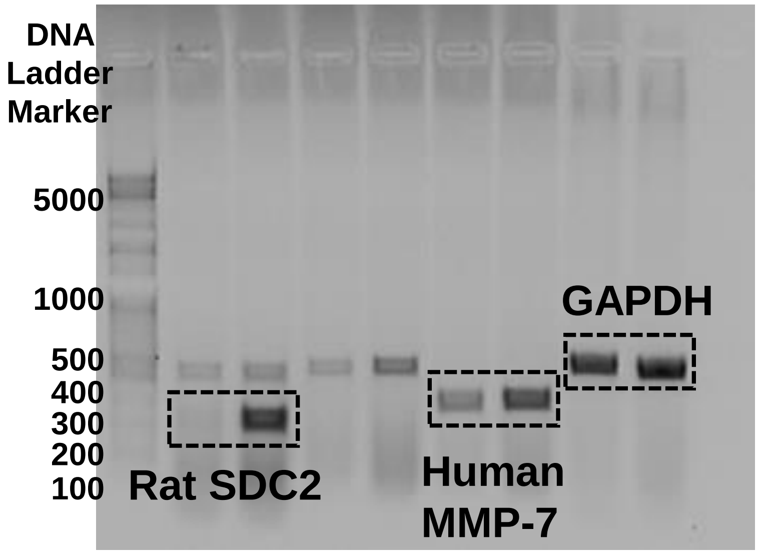

B

Original gels presented in Figure 3C

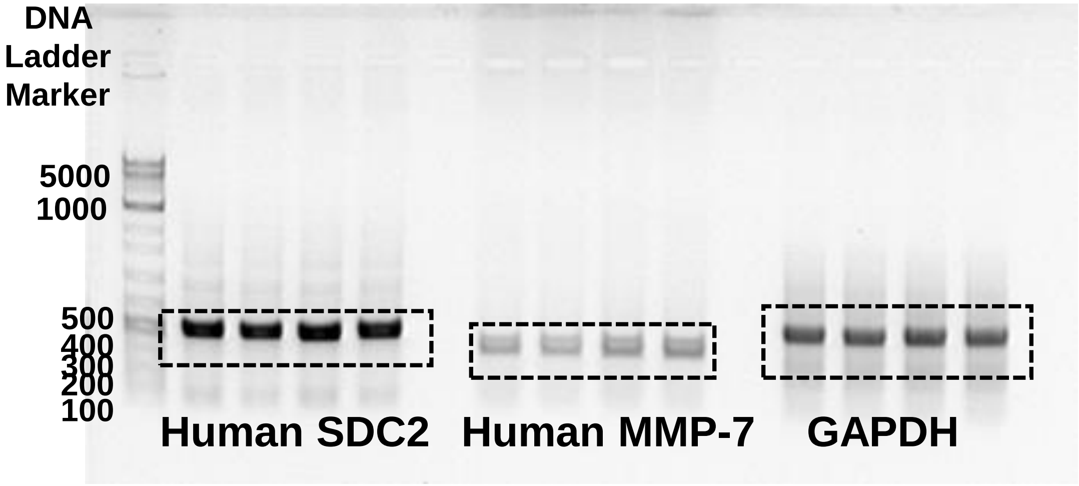

Rat SDC2
product length = 283

Human MMP-7
product length = 373

Human SDC2
product length = 540

GAPDH
product length = 598

Figure S2

Full sequence of pro-domain of MMP-7 used for fluorescence assay

NH2-MLPLPQEAGGMSELQWEQAQDYLKRFYLYDSETKNANSLEAKLKEMQKFFGLPITGMLNSRVIEIMQKP
RCGVPDVASY-COOH

Sequence alignment between human and rat SDC2

```
Human SDC2 ---------------------------------------MYLDNSSIEEASGVYPIDDDD 21
Rat SDC2   MRVRATSPGNMQRAWILLTLGLMACVSAETRAELTSDKDMYLDSSSIEEASGLYPIDDDD 60
                                                  ****.********:*******

Human SDC2 YASASGSGADEDVESPELTTSRPLPKILLTSAAPKVETTTLNIQNKIPAQTKSPEETDKE 81
Rat SDC2   YSSASGSGAYEDKGSPDLTTSQLIPRISLTSAAPEVETMTLKTQSITPTQTESPEETDKK 120
           *:******* **  **:****: :*:* ******:*** **: *.  *:**:*******:

Human SDC2 KVHLSDSERKMDPAEEDTNVYTEKHSDSLFKRTEVLAAVIAGGVIGFLFAIFLILLLVYR 141
Rat SDC2   EFEISEAEEKQDPAVKSTDVYTEKHSDNLFKRTEVLAAVIAGGVIGFLFAIFLILLLVYR 180
           :..:*::*.* *** :.*:********.********************************

Human SDC2 MRKKDEGSYDLGERKPSSAAYQKAPTKEFYA 172
Rat SDC2   MRKKDEGSYDLGERKPSSAAYQKAPTKEFYA 211
           *******************************
```
